# EEG Emotion Recognition Applied to the Effect Analysis of Music on Emotion Changes in Psychological Healthcare

**DOI:** 10.3390/ijerph20010378

**Published:** 2022-12-26

**Authors:** Tie Hua Zhou, Wenlong Liang, Hangyu Liu, Ling Wang, Keun Ho Ryu, Kwang Woo Nam

**Affiliations:** 1 Department of Computer Science and Technology, School of Computer Science, Northeast Electric Power University, Jilin 132000, China; 2 Data Science Laboratory, Faculty of Information Technology, Ton Duc Thang University, Ho Chi Minh City 700000, Vietnam; 3 Biomedical Engineering Institute, Chiang Mai University, Chiang Mai 50200, Thailand; 4 Department of Computer and Information Engineering, Kunsan National University, Gunsan 54150, Republic of Korea

**Keywords:** EEG signals, emotion recognition, music therapy, semantic analysis

## Abstract

Music therapy is increasingly being used to promote physical health. Emotion semantic recognition is more objective and provides direct awareness of the real emotional state based on electroencephalogram (EEG) signals. Therefore, we proposed a music therapy method to carry out emotion semantic matching between the EEG signal and music audio signal, which can improve the reliability of emotional judgments, and, furthermore, deeply mine the potential influence correlations between music and emotions. Our proposed EER model (EEG-based Emotion Recognition Model) could identify 20 types of emotions based on 32 EEG channels, and the average recognition accuracy was above 90% and 80%, respectively. Our proposed music-based emotion classification model (MEC model) could classify eight typical emotion types of music based on nine music feature combinations, and the average classification accuracy was above 90%. In addition, the semantic mapping was analyzed according to the influence of different music types on emotional changes from different perspectives based on the two models, and the results showed that the joy type of music video could improve fear, disgust, mania, and trust emotions into surprise or intimacy emotions, while the sad type of music video could reduce intimacy to the fear emotion.

## 1. Introduction

Emotional influence is a highlight issue in modern life, and positive emotions can promote individuals’ health and work efficiency [1]. In daily life, people are listening to different types of music in different emotional states, and it has been proven that music can arouse the audience’s emotions and change their emotions [2]. The music therapy application scope is more extensive than simple entertainment and pleasure, especially for psychological healthcare in society. In recent years, music moods recognition and music emotional effects analysis have been highlighted issues, which annotate emotional tags in combination with typical music features through data mining and machine learning technology [3]. As an application in the field of music emotion, music therapy is considered the most promising technology to help to adjust emotions [4].

Music therapy related to EEG emotion semantic analysis as a new research hotspot, which focuses on physiological information analysis, where a large amount of information related to emotional stated can be retrieved [5]. Among the physiological signals, the EEG can directly and effectively detect a real emotional state based on brain activities [6]. Generally, EEG wavelet signals are always divided into five different frequency bands according to the frequency range, including 1–4 Hz (*δ*), 4–8 Hz (*θ*), 8–16 Hz (*α*), 16–32 Hz (*β*), and 32–64 Hz (*γ*) [7].

Emotion is a physiological feeling that relates to various thoughts and behaviors. Plutchik [8] constructed an eight basic emotion types wheel, and believed that other complex emotions could be formed from the combination of these basic emotions. Lang [9] proposed an emotion model based on a two-dimensional coordinate system with valence and arousal.

Related research shows that EEG emotion semantic analysis is effective. Ismail et al. [10] studied the four emotions of happiness, sadness, surprise, and anger using 32 EEG channels. The results showed that there was a theta reaction in the right side of the brain when angry; a delta and theta reaction in the back-right of the brain when sad; an alpha reaction in the middle of the brain when happy; and delta and theta reactions for surprise. Hu et al. [11] studied the relationship between 10 positive moods. According to the participants’ similarity evaluation, there were three further categories, and emotion recognition was carried out. The accuracy rate was above 80% using 32 EEG channels. Gao et al. [12] proposed an EEG signal feature with multi-scale information analysis (MIA). In this study, EEG signal high-frequency bands were more related to emotion expression than low-frequency bands, and the temporal and frontal regions appeared to be significant response areas. Li et al. [13] conducted emotion recognition in a two-dimensional emotion model with an EEG and DEAP (emotion and videos) dataset. It appears that the γ band showed higher classification accuracy than the others. Moreover, Ahmed et al. [14] considered a deep learning model to classify the emotion types based on Asmap, which labeled brain regions in a 2D vector. And Khateeb et al. [15] presented a multi-domain feature fusion model to classify nine kinds of mood types. They both used the DEAP dataset and discussed the multiple types of emotions that were recognized.

Regarding studies of music, most of them are about the music genre, and the analysis of music emotion is featured relatively less. Kostiuk et al. [16] combined audio and video features to more effectively recognize emotions in the overextended CAL500 database. Gkaitatzis et al. [17] analyzed the influence of different music on emotions using 32 EEG channels and found that listening to 120 BPM music was satisfactory and pleasant, while music that was faster than 170 BPM and slower than 70 BPM made people feel more stressed and unhappy. Bai et al. [18] analyzed four types of music emotion: happy, angry, sad, and relaxed. They extracted 548-dimensional music features, and the accuracy rate was more than 80%. Xu et al. [19] proposed a source separation technology for four music emotion type recognitions, namely, anger, calm, happiness, and sadness. The experimental results showed that the source separation technology was helpful to identify music emotion, accompaniment music had a better recognition effect than singing, and performance was improved through later fusion.

At present, using EEG emotion recognition technology for disease treatment, such as cancer and heart related diseases, has offered a new research hotspot. Moreno et al. [20] proposed a medical intelligent system using PSD based on the EEG band to measure the effects of dolphin therapy. Kinugasa et al. [21] applied a music therapy system by using EEG to estimate emotions. The system evaluated emotions through EEG and presented music that best matched an emotion. The overall accuracy of the experimental results was about 60%. McConnell et al. [22] developed a device to produce appropriate music through physiological signals. In addition, the emotional state was evaluated using physiologically objective information for music therapists about users in real time. EEG recognition has also been widely used in ML-based diseases [23,24,25], as well as mental workload predictions in a broad scope [26]. Hussain et al. [26] mentioned mental workload induced by various driving environments issue, and proposed an EEG-based neurological state prediction approach that could be utilized in an advanced driver assistance system (ADAS). Ramirez et al. [27] studied treatment effects for cancer patients using music therapy, which focused on the 32 EEG channels’ emotional recognition capacities. In fact, positive emotion changes using music therapy seemed to be effective for advanced cancer patients. They also reduced fatigue, anxiety, and dyspnea, and increased happiness, based on results from a questionnaire survey.

Although mainstream studies have proven that EEG based emotion recognition is effective and feasible, a large number of channels are still used for emotion recognition. However, there are little research on the combination of EEG and music, and the influence of music on emotions needs further study.

Our contributions is a study of emotion recognition from EEG and audio signals, coupled with an analysis using music therapy. We used EEG signals to identify the user’s emotional state and optimize the channel selection, and then combined the emotional elements and music classification to achieve the emotional classification of the music. Finally, through the research and analysis of the relationship between music emotional types and emotional state changes, we provided a reference for the selection of music in music therapy. The rest of this paper is organized as follows: materials and detailed methods are presented in Section 2; Section 2.2 describes the emotion recognition model based on EEG signals; Section 2.3 describes the emotion classification model based on audio signals; the experimental results and evaluation are presented in Section 3; related discussion and future work are presented in Section 4; and, finally, the conclusion of this work is detailed in Section 5.

## 2. Materials and Methods

In music therapy, it is difficult to effectively improve the emotional state by simply choosing some positive music. For example, in music therapy, listening to happy music does not necessarily make people feel happy in a sad state. For the music selection, more experimental research is needed to obtain the theoretical basis of music selection. There needs to be a focus on the analysis of the relationship between emotional changes, as well as the influence of different types of emotional music on emotional state and emotional changes, which can provide a reference for music selection in the experiment. Using music data with genre labels cannot achieve the purpose of studying emotional relationships in music therapy experiments. In order to provide music data with emotional labels for music therapy experiments, we integrated emotional elements into music classification, as well as studied and analyzed music-based emotion classifications. In addition, considering the portable EEG acquisition equipment requirement, we optimized the number of EEG channels based on a semantic related model, which can also reach a higher accuracy.

Therefore, this paper first studied EEG emotional semantic recognition and analyzed 20 kinds of emotions through emotion mapping. On this basis, we analyzed the emotion recognition of each channel and optimized the channel selection according to the accuracy of each channel so as to achieve reliable emotion recognition based on fewer EEG channels for portable mobile applications. Then, we combined emotional elements with music classification, studied the emotion classification based on the audio signal, and classified eight kinds of music to provide emotion tags for music data. Finally, in the music therapy experiment, we focused on the analysis of the relationship between emotional changes and the impact of different types of emotional music on the user’s emotional state, and we provided a reference for the music therapy experiment based on the obtained relationship for emotional transformation.

**Figure 1:**
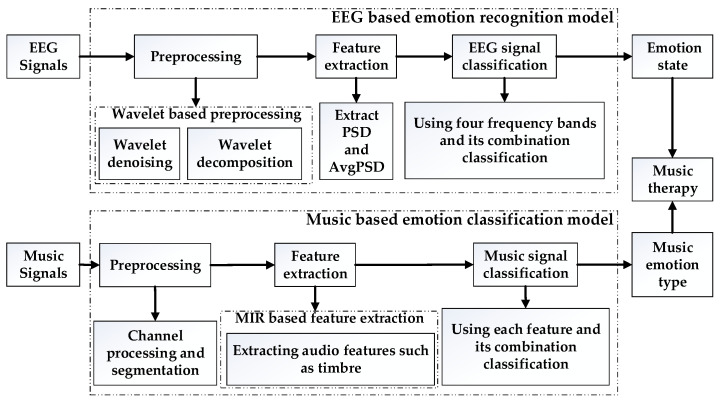
General architecture of music therapy calculation model.

The general architecture is shown in Figure 1. The whole process contained two main models—the EER model and the MEC model. The EER model was used for recognizing EEG signals, which were related to 20 different kinds of emotions by extracting the average PSD features. The MEC model was used for recognizing music signals, which were also related to emotions by calculating the audio features and classifying the music emotion types. Then, we combined the EEG emotion state and music emotion type to perform deep analysis of the music therapy efficiency in order to mine the rules of moods changes after listening to different types of emotional music and know about the real emotion changes by detecting the EEG emotion changes. 

### 2.1. Datasets

The DEAP dataset included the EEG and peripheral physiological signals of 32 participants who watched 40 music video clips, and the EEG data was acquired from 32 channels. For details on the DEAP dataset, see [28]. DREAMER is a multi-modal database consisting of electroencephalogram (EEG) and electrocardiogram (ECG) signals recorded during affect elicitation by means of audio-visual stimuli. For details on the DREAMER dataset, see [29]. We collected music data and label data from last.fm and Spotify. We considered the following eight emotions, ‘fear’, ‘confidence’, ‘joy’, ‘anger’, ‘disgust’, ‘passion’, ‘relaxation’, and ‘sadness’, and build a dataset of 356 songs. In our test, we considered these three types of datasets and gave the experiments results in Section 4. 

### 2.2. EEG Emotional Recognition Model (EER Model)

**Figure 2:**
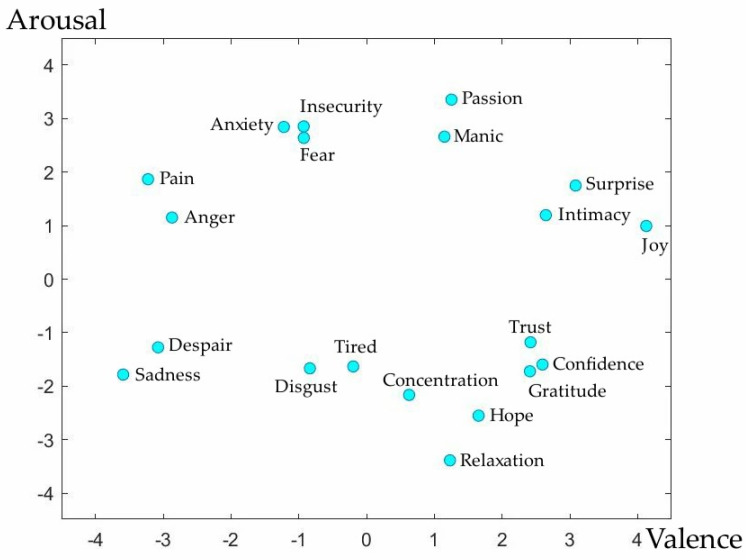
The 20 emotions in the valence arousal model.

Our proposed EER model was used for identifying the multi-dimension emotional changes to evaluate music therapy efforts, which could objectively reflect real mood changes. In our previous study, we studied a lot of microblog social big data to train the core emotion types for modern people. There were 20 typical core emotions derived by calculating the similarity and angle [30]. Moreover, we tested the real EEG signals based on these 20 emotion types, which exactly appeared with significant arousal rates [31]. After semantic distance calculating, Figure 2 shows these 20 kinds of emotions’ distributions in the valence arousal model. Then, we annotated emotion labels in the DEAP dataset based on the valence arousal model. Actually, these 20 typical emotion labels were related to more than ten thousand emotions by calculating the semantic similarity distance between these 20 typical emotion labels based on our own constructed sentiment dictionary. That is why our proposed model could achieve higher recognition accuracy than the traditional methods. These 20 emotions include intimacy, surprise, tiredness, anxiety, concentration, pain, relaxation, confidence, disgust, insecurity, fear, despair, hope, gratitude, anger, passion, sadness, mania, joy, and trust. The distribution of the 20 emotions in the two-dimensional model is shown in Figure 2.

Usually, EEG original signals contain many kinds of noise, such as white Gaussian noise, artifacts caused by muscle movement [32], and eye blink artifacts [33]. In this study, we focused on solving Gaussian noise, and selecting the wavelet denoising threshold was a core preprocessing approach. The extremum threshold estimation method can extract the weak signal with a better effect, while the fixed threshold estimation and the heuristic threshold estimation denoise more thoroughly. It also easier to judge the useful signal into noise data, although the denoising process is more effective. So in this study, we selected the mother function of Daubechies (db4) to remove noise, and the maximum wavelet decomposition level was adopted and calculated to the suitable threshold values based on the extremum threshold estimation rule, which is shown in the following equation:

(1) $$\lambda =\{\begin{array}{c} \partial \left(0.3936+0.1829\left(\frac{lnE}{ln2}\right)\right),E>32\\ 0,E\leq 32 \end{array}$$

where *E* denotes the signal length, *λ* denotes the threshold value, and *∂* denotes the noise variance coefficient. The noise variance equation is as follows:

(2) $$\partial =\frac{M_{w}}{0.6745}$$

Parameter *w* denotes the wavelet coefficient, $M_{w}$ is the median of the wavelet coefficient vector, and ∂ denotes the noise variance coefficient. The denoising equation is as follows:

(3) $$d=\{\begin{array}{c} w,\;\left|w\right|\geq \lambda \\ 0,\;\left|w\right|<\lambda \end{array}$$

where *d* denotes the wavelet coefficient after noise reduction, *w* denotes the wavelet coefficient before noise reduction, and λ is a threshold. 

#### 2.2.1. Feature Extraction

Because wavelet transformation is widely used for solving nonstationary and nonlinear datasets, it was suitable for calculating EEG signals. So, in this case, EEG signals were decomposed by wavelet transformation, and then every frequency band’s power spectral density was extracted as reference features. The sub-band frequency range calculation based on the wavelet decomposition principle and frequency of signals is shown in Equation (4):

(4) $$\left[1,\frac{\mathrm{fs}}{2^{5}}\right],\;[\frac{\mathrm{fs}}{2^{5}},\frac{\mathrm{fs}}{2^{4}}],\;[\frac{\mathrm{fs}}{2^{4}\;},\;\frac{\mathrm{fs}}{2^{3}}],\;[\frac{\mathrm{fs}}{2^{3}},\frac{\mathrm{fs}}{2^{2}}],\;[\frac{\mathrm{fs}}{2^{2}},\frac{\mathrm{fs}}{2}],\;[\frac{\mathrm{fs}}{2},128]$$

Parameter *fs* denotes the sampling frequency, and *[a,b]* denotes the frequency range from *a* to *b* for each level.

In the current research, wavelet decomposition should choose the same wavelet function as wavelet denoising. So, the db4 mother wavelet was decomposed by five levels according to Equation (4). The detailed information of the decomposition level, frequency range, and corresponding frequency band is shown in Table 1. 

When applying the 4.0–45.0 Hz bandpass frequency filter, the delta rhythm is traditionally regarded as being correlated with sleep and is not considered further in our study. So, we only considered four kinds of frequency bands, *θ* (4–7 Hz), *α* (8–13 Hz), *β* (14–30 Hz), *γ* (31–50 Hz), to calculate the PSD features. We used the Welch periodogram method to calculate the PSD Pxx signal, as is seen in Equation (5). Given that the EEG signal cannot be exactly divided into eight parts, a Hamming window was added to each part with an overlap ratio over 50%. The signal was automatically clipped accordingly.

(5) Pxx= pwelch (x)

Parameter *x* is the original signal, *pwelch* is a function of power spectral density based on the Welch periodogram method, and *Pxx* denotes PSD values.

After calculating the PSD of each frequency band, we calculated the sum of the PSD and then divided that total by the PSD length to average the PSD. In addition, we used a logarithmic function to process the data, so as to limit the excessive power for a certain frequency band, and its features were in a specific order magnitude, as Equation (6) shows. The above work was carried out for the four respective frequency bands, and, finally, the average PSD value of each kind of frequency band was obtained as the feature.

(6) $$AvgPSD=\frac{\sum_{i=1}^{P}10*log10\left(Pxx_{i}\right)}{P}$$

where $Pxx_{i}$ denotes the ith point of power spectral density *Pxx*, *P* denotes the length of the power spectral density *Pxx*, and AvgPSD denotes the average power spectral density.

Since the DEAP data set contains 32 channels of EEG signals, experiments were conducted based on multi-channel EEG signals. For details on the DEAP data set, see Section 3.1. In order to optimize the channel used in the experiment, we first conducted experiments based on each channel of the 32 channels to obtain the accuracy of each channel. Then, we sorted the 32 channels according to the accuracy of each channel using the selection sort method. The equation is as follows:

(7) $$List=\mathrm{sorting}\left(acc_{channel}\right)$$

where $acc_{channel}$ denotes the accuracy of each channel, sorting denotes the sorting function, and *List* denotes the order list of channels after sorting.

**Figure 3:**
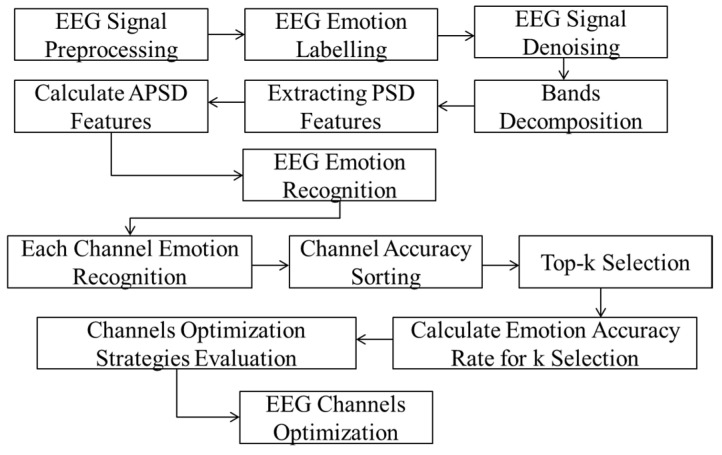
EEG-based emotion recognition model flow work.

According to the order of channels, the best two channels, three channels, and four channels were selected to calculate and compare the accuracy for each manner. The final channel selection was determined by the result of the analysis, so as to use fewer EEG channels to also guarantee the emotion identification accuracy. The purpose was to carry out reliable emotion recognition on portable devices in the future. The whole EEG-based emotion recognition model processing is shown in Figure 3:

#### 2.2.2. EEG-based Emotion Recognition Algorithm

The EER algorithm was more efficient for identifying these 20 kinds of emotions based on extracted EEG features, and the defined parameters are shown as follows (Table 2):

The detailed EER Algorithm 1 (* The best −*c* and −*g* are obtained by the grid search method and 10−fold cross validation.) was defined as follows:
**Algorithm 1:** EER Algorithm**Input:** ES **Output:** maximum Acc and average Acc 1: **Begin** 2: import ES to Matlab 3: *X ← ES; TPTR ← minimaxi; WNAME ← db4;* 4: **for** each of ES **do** 5:  WDS = wavelet denoising(X, ‘TPTR’, ‘wname’); 6: **end for** 7: *X ← WDS; WDL ← 5; wname ← db4;* 8: **for** each of WDS **do** 9:  FBS = discrete wavelet transforms(X, N, ‘wname’); 10: **end for** 11: **for** each of FBS **do** 12:  Pxx = pwelch(FBS); 13:  AP = sum(10*log10(Pxx))/length(Pxx); 14: **end for** 15: **for** AP of each ES **do** 16:  *−t = 2*
17:  **for** 10-fold cross validation **do**
18:   get best parameter *−c* and parameter *−g* 19:  **end for** 20:  cmd = [*‘ −t’,’ −c ‘, ‘ −g ‘*] 21:  model = libsvmtrain (*train_label, train_data, cmd*) 22:  classification accuracy as Acc = libsvmpredict (*test_label, test_data, model*) 23: **end for**

### 2.3. Music-Based Emotion Classification Model (MEC Model)

The MEC model was used to identify music emotions, which signified most of the listeners’ emotional feelings after listening to different kinds of music. Through this model, audio features, such as timbre, rhythm, and intensity, can be extracted from the music data. There were eight types of music data that were obtained, and then the left and right channels of audio signals were merged and segmented to keep the balance of the dataset. We combined the left and right channel signals of music to achieve the purpose of retaining all audio information. Then, we empirically set the length of a clip to be 15 s, which resulted in 5932 clips in total, as is shown in Table 3.

#### 2.3.1. Audio Features Extraction

Then, we extracted features from audio data in order to obtain better performance for the music classification. Different sets of features were extracted that represented different features of the music cue. Mel frequency cepstral coefficients (MFCCs) represent the music frequency content’s effect on the human hearing system. The MFCC equation is as follows:

(8) $$\mathrm{MFCC}=\overset{N-1}{\underset{m=0}{\sum }}E\left(m\right)\cos\left(\frac{\pi n\left(m-0.5\right)}{M}\right),n=1,2,\ldots ,L$$

Parameter *N* is the sampling points number, *L* is MFCC coefficient order, *M* is the triangular filters number, and *E* (*m*) is the logarithmic energy of the output of the filter bank.

Timbre features are considered to represent the spectral shape of the music cue. The spectral centroid equation is as follows:

(9) $$\mathrm{Spectral}\;\mathrm{Centroid}=\frac{\sum_{n=1}^{F}fs(n)*E(n)}{\sum_{1}^{F}E(n)}$$

Parameter *F* denotes the highest signal frequency, *fs (n)* denotes the signal frequency, and *E (n)* denotes the spectral energy of the corresponding frequency after the short-time Fourier transformation of the continuous time domain signal x (T). The spectral flux equation is as follows:

(10) $$\mathrm{Spectral}\;\mathrm{Flux}=\sum_{k=1}^{K}{\left(N_{i}\left(k\right)-N_{i-1}\left(k\right)\right)}^{2}$$

Parameter *K* denotes discrete Fourier transform coefficients number, and $N_{i}\left(k\right)$ denotes the *k*th normalized discrete Fourier transform coefficient of the *i*th frame.

One of the temporal features of acoustic cues is the zero crossing rate. The zero crossings equation is as follows:

(11) $$\mathrm{Zero}\;\mathrm{Crossings}=\frac{1}{2}\overset{M-1}{\underset{m=0}{\sum }}\left|\mathrm{sign}\left[x_{m}\right]-\mathrm{sign}\left[x_{m-1}\right]\right|$$

where *x* denotes the signal, *M* denotes the length of the signal, and *sign* denotes a sign function, which is as follows:

(12) $$\mathrm{sign}\left[x\right]=\{\begin{array}{c} 1,x\geq 0\\ -1,x<0 \end{array}$$

where *x* denotes the value entered; when *x* is equal to or greater than 0, the value is 1; when *x* is less than 0, the value is –1.

Intensity features represent the energy content of the music cue. The root mean square equation is as follows:

(13) $$\mathrm{Root}\;\mathrm{Mean}\;\mathrm{Square}=\sqrt{\frac{\sum_{i=1}^{M}X_{i}^{2}}{M}}$$

where *M* is signal length, and $X_{i}^{2}$ is the *i*th signal square value.

Rhythm is one of the most basic features of music cues. Different rhythms make listeners experience various emotional states. The rhythm features of music can be obtained by extracting the beat and the strength of the beat. The beat per minute (BPM) equation is as follows:

(14) $$\mathrm{BPM}=\frac{60}{T}$$

where *T* denotes the time interval between each beat in seconds. The BPM intensity is extracted by using the Matlab function “mirpulsecularity”.

Features related to modes are used to estimate if the piece is in the major, or in the minor mode, so as to achieve different emotional constructions in music science. The harmony is extracted by using the Matlab function “mirmode”. The roughness is a measurement of sensory dissonance. Stranger oscillations will lead to the sounds feeling harsher, and the roughness value will be higher. The roughness equation is as follows:

(15) $$\mathrm{Roughness}=\sum_{i=1}^{i=24}\frac{r\left(e_{i}+e_{i-1}\right)}{2}$$

where *e* denotes the envelope time function coefficient of the characteristic frequency band, and *r* denotes the spectral density of the roughness. 

MFCCs (Mel frequency cepstral coefficients) represent the influence of the frequency content of music on the human hearing system. Timbre features are considered to represent the spectral shape of the music cue. One of the temporal features of acoustic cues is the zero crossing rate. Intensity features represent the energy content of the music cue. Rhythm is one of the most basic features of music cues. Different rhythms make listeners experience various emotional states. The rhythm features of music can be obtained by extracting the beat and the strength of the beat. Features related to mode are used to estimate if the piece is in the major, or in the minor mode, so as to achieve different emotional constructions in music science. 

For each clip, we extracted a combined 21-dimensional feature vector using the MIR-toolbox. These features include timbre, temporal, intensity, rhythm, harmony, and other audio features. The detailed information of the extracted audio features is shown in Table 4.

The music emotion classification (MEC) algorithm can efficiently extract the audio features from the music signals and classify eight kinds of emotions in music. The parameters that defined the algorithm are listed in Table 5.

#### 2.3.2. Music Emotion Classification Algorithm

The detailed MEC Algorithm 2 (* The best −*c* and −*g* are obtained by the grid search method and 10−fold cross validation) was defined follows:
**Algorithm 2**: MEC Algorithm**Input:** MS **Output:** maximum Acc and average Acc 1: **Begin** 2: import MS to Matlab 3: read the left and right channel signals of MS and merge them 4: **for** each of MS **do** 5:  SN = fix(length(MS)/44,100/15); 6:  **for** *i* = 0, *i* < SN – 1, *i* ++ **do** 7:   SST = i * 44,100 * 15 + 1; 8:   SEN = (i + 1) * 44,100 * 15; 9:   SS= audio signal (SST:SEN); 10:  **end for** 11: **end for** 12: **for** each of SS **do** 13:  Calculate 9 kinds of music features and form MFV 14: **end for** 15: **for** MFV of each MS **do** 16:  *−t = 2* 17:  **for** 10 fold cross validation **do** 18:   get best parameter *−c* and parameter *−g* 19:  **end for** 20:  cmd = [*‘ −t’, ‘−c’, ‘−g’*] 21:  model = libsvmtrain (*train_label, train_matrix, cmd*) 22:  classification accuracy as Acc = libsvmpredict (*test_label, test_matrix, model*) 23: **end for**

### 2.4. Emotion Awareness based on EER and MEC Models

**Figure 4:**
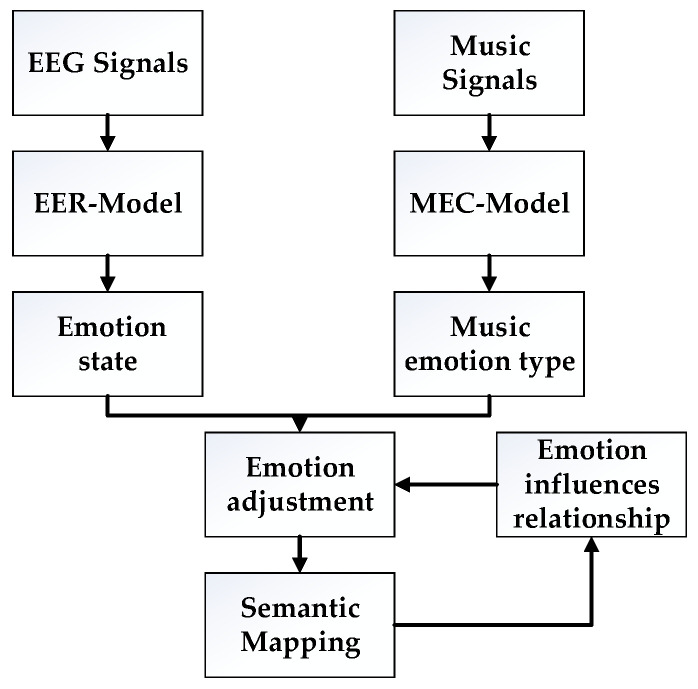
Emotion awareness based on EER and MEC models.

The EER model identified emotions based on EEG signals to support the detection of emotional states, and the MEC model classified music based on audio signals to provide emotional tags for music. In this section, we carried out the emotion adjustment by combining the above two models, as is shown in Figure 4.

Firstly, the EEG signals of users were collected and analyzed using the EER model to obtain the emotional state of users. At the same time, music data were collected and analyzed using the MEC model to obtain the emotion type tags of the music. Secondly, the emotion adjustment experiment was carried out, and the semantic mappings were analyzed to mine the potential correlations between emotional transformations and music emotions. Finally, the reliability of emotion adjustment was improved according to the emotion influence relationship obtained by emotion mapping.

We analyzed emotional semantic mapping from different perspectives. Firstly, the relationship between 20 kinds of emotional states and 8 kinds of music emotions was studied. Secondly, in taking the type of music emotion as a quantitative study, the influence of music emotion on emotional change was analyzed. Finally, we analyzed the relationship between music emotion types and emotional changes based on the valence arousal model. In addition, we designed an experiment to verify the above contents, and the specific analysis results are shown in Section 3.4.

## 3. Experiment and Results

The computer CPU used in the experiment was Xeon 2620, the main frequency was 2.1GHz, and the software was MATLAB2020a. We considered MIRtoolbox [34] to experiment and adopted LIBSVM for classification [35].

### 3.1. Datasets

In our experiments, we considered three datasets: the DEAP dataset [28], the DREAMER dataset [29], and emotional music datasets. For the detailed information, see Section 2.1.

### 3.2. EER Model Tests

**Figure 5:**
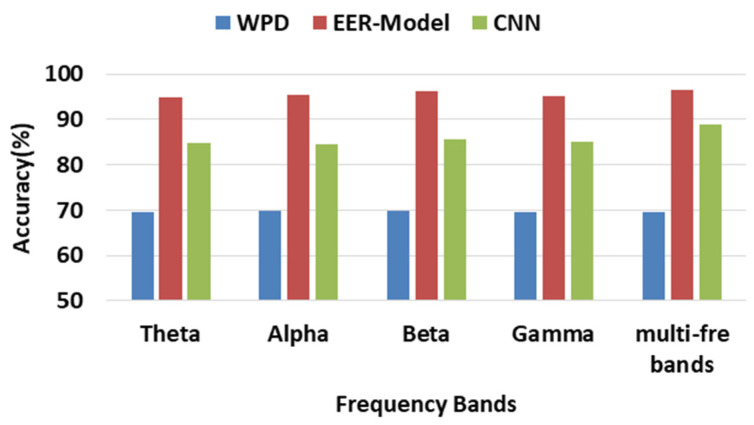
Average accuracy comparison among WPD, CNN, and the EER model.

The EER model is efficient for recognizing different kinds of emotions based on EEG signals. We separately calculated the accuracy rate for each band and combined some of the related bands together to evaluate their accuracy for classification. The combination of four frequency bands was named ‘multi-frequency bands,’ and the classification results based on 32 channels using the DEAP dataset is shown in Figure 5. Our proposed EER model’s accuracy rate reached 96.47%, which was achieved by using the combination of each frequency band. We also compared our model with the wavelet packet decomposition (WPD) algorithm [36], and its classification accuracy rate reached 69.74% by using the beta band. We also used convolutional neural networks (CNN) for classification, and the accuracy of *θ*, *α*, *β*, *γ*, and the multi-frequency bands was 84.88%, 84.68%, 85.51%, 85.08%, and 88.81%, respectively. At the same time, we also used the DREAMER as comparison datasets. The *θ*, *α*, *β*, *γ*, and multi-frequency bands accuracy were 79.76%, 76.09%, 83.07%, 80.11%, and 82.37%, respectively.

**Figure 6:**
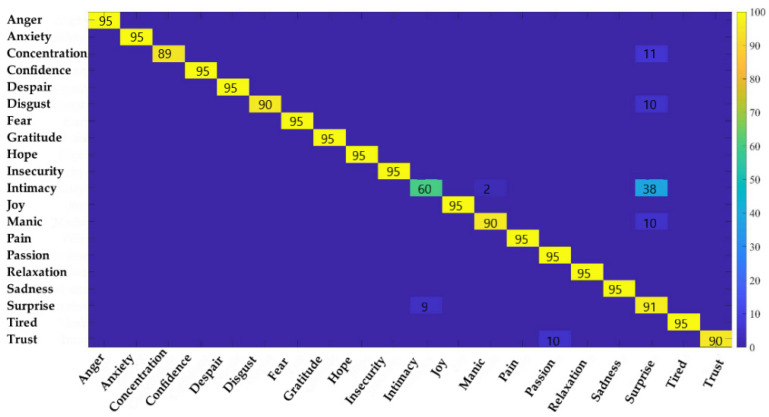
Confusion matrix of twenty emotions using the DEAP dataset.

We used the confusion matrix to discuss the frequency band combination identification effects analysis, as is shown in Figure 6. It was found that there was a large recognition error between intimacy and surprise. The accuracy rate for intimacy was only 60%, and 38% of the probability prediction was surprising, which also showed that the two emotions are more similar.

In addition, in considering the further portability of emotion recognition, we carried out emotion recognition for each frequency band of each channel, and the accuracy of emotion recognition using a single-band, single-channel EEG signal was only 10% to 20%, while the accuracy of emotion recognition using the four bands combination reached 40% to 60%, though this was still not ideal. By observing that the recognition accuracy of each channel was different, we made further analysis.

**Figure 7:**
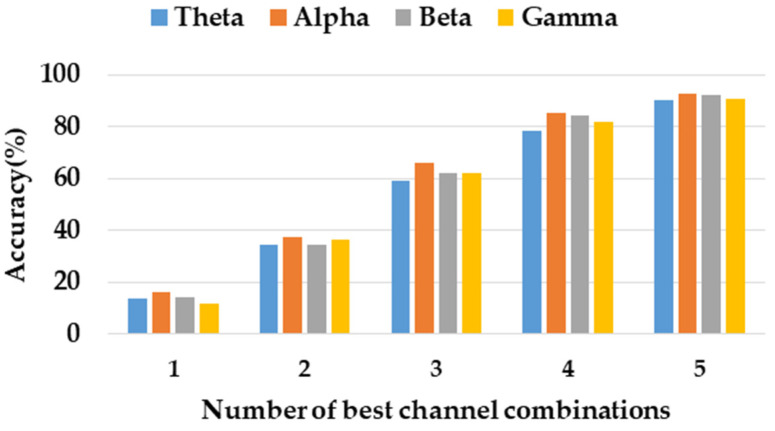
Average accuracy comparison of best channel combination options.

In considering the further portability of emotion recognition, we studied the minimum number of channels to identify emotions while maintaining high accuracy. According to the accuracy rate of each channel, we sorted and selected the five best channels. From high to low, the channel names were P7, FC1, PZ, T7, and CP2. Then, we combined them according to the best channel order, and the recognition results of each frequency band are shown in Figure 7. When the best three channels were used, the accuracy rate of each frequency band could reach about 60%, and when the best five channels are used, the accuracy rate of each frequency band could reach about 90%.

### 3.3. MEC Model Tests

In this section, we tested music emotion classification accuracies with our proposed MEC model, and discussed the relationship between music features and emotion changes. We extracted the spectral centroid, spectral flux, zero crossing rate, intensity, rhythm, rhythm intensity, harmony, and roughness feature of the eight kinds of music and calculated the average value of each feature of the eight kinds of music. The statistical results are shown in Table 6. By observing and comparing the features of the eight kinds of music, it was found that the rhythm intensity, tone, and roughness for anger and fear were similar; the spectral centroid, spectral flux, zero crossing rate, intensity, rhythm and rhythm intensity for joy and passion were similar; the values of each feature of confidence and disgust were relatively high, and disgust was the highest. The values of each feature of relaxation and sadness were relatively low, and relaxation was the lowest.

**Figure 8:**
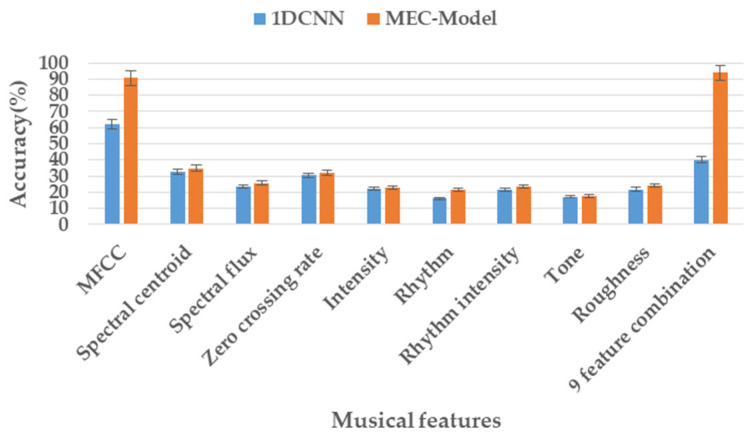
Audio features average accuracy comparison.

We further discussed the most related music features with higher classification accuracies for emotions, as is shown in Figure 8. For each kind of audio feature and the nine feature combination, the accuracy rate was 90.76%, 34.85%, 25.72%, 31.77%, 22.78%, 21.24%, 23.43%, 17.73%, 24.11%, and 94%, respectively. The results showed that the MFCC features were the best, and the classification accuracy reached 90.76%, which was far higher than other features. In addition, we combined the other eight features, except for MFCC features, and the classification accuracy was also very high at 83.73%; however, this was still not as accurate as using MFCC features alone. Finally, the classification accuracy reaches 94% by combining all the features. In addition, we classified using the 1-dimension convolutional neural networks (1DCNN) algorithm as a comparison, and the MFCC features achieved the best classification result with an average accuracy rate of 62.05% [37].

**Figure 9:**
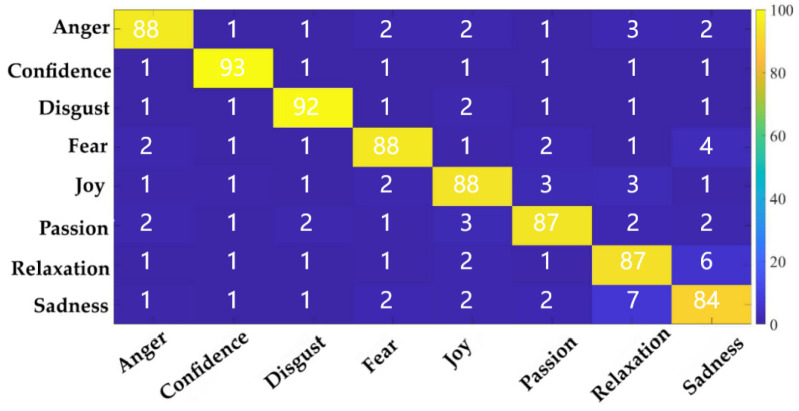
Confusion matrix of eight kinds of music.

We used confusion matrix to evaluate the feature combination, as is shown in Figure 9. It was found that some predicted labels were wrong, and there was a large prediction error between relaxation and sadness. There was a 10% chance that these two classes would be mistaken for each other, which also showed that the two music types were more similar. In addition, according to the statistical analysis of the features, the above conclusions could also be explained.

In addition, we used the music used in the DEAP dataset to verify the MEC model. The accuracy of joy, passion, sadness, and relaxation were 80%, 50%, 100%, and 67%, respectively. At the same time, we considered that our model was intended to classify the music of the 15 s segment, but the music label of the DEAP dataset was the overall label of the music, and there was a deviation between the label of every 15 s segment and the overall label, so we combined the tags of music segments into a tag sequence, and then analyzed the overall label of music in the valence arousal model. The results showed that the accuracy of the HVHA, LVHA, LVLA, and HVLA was 70%, 50%, 70%, and 20%, respectively. Then, according to the classification results of the LVHA and HVLA and the emotional label distribution of the DEAP music dataset, it was found that most of the music with the wrong classification was at the junction of quadrants, which was easy to identify as the emotion of adjacent quadrants.

### 3.4. Music Therapy Analysis

**Figure 10:**
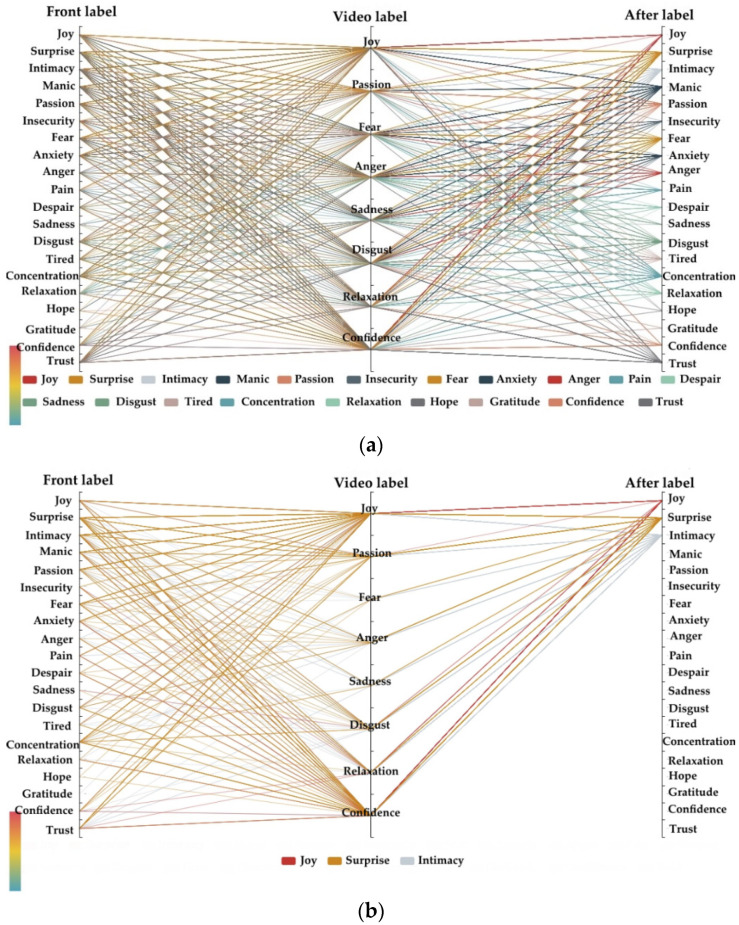
Emotion parallel graph. (**a**) 20 emotions; (**b**) parallel graphs of joy, surprise, and intimacy.

To perform deep analysis of the potential rules of emotional changes before and after listening to music based on the EER model and MEC model, a parallel coordinate map was drawn in Figure 10, which, according to the EEG appearance, real emotional tags changed before and after with different kinds of emotional music video interventions.

Figure 10a is a graph of all 20 kinds of emotions. Due to the display of all emotions being too complicated, we simplified it according to “After label” to display only a part of the emotions. For example, most of the emotions could be improved into joy, surprise, and intimacy emotions through music videos of joy and confidence, which were similar in the two-dimensional emotional model and were all positive valence, as is shown in Figure 10b. In addition, manic emotions were mainly caused by fear, anger, and disgust in music videos. The anxiety emotion was mostly caused by anger type music videos. Disgust and tired emotions were mainly caused by disgust and sadness in music videos.

By taking music video type as quantitative, the influence of music videos on emotional change was observed, and we selected some data with high probabilities, as is shown in Table 7. The confidence type music video could promote concentration, disgust, and fear emotions to surprise with an increased probability of more than 41%. The joy type of music video could improve fear, disgust, mania, and trust emotions into surprise or intimacy emotions, and the improvement probability was more than 44%. The sad type of music video could reduce intimacy to the fear emotion with a probability of 46%.

**Figure 11:**
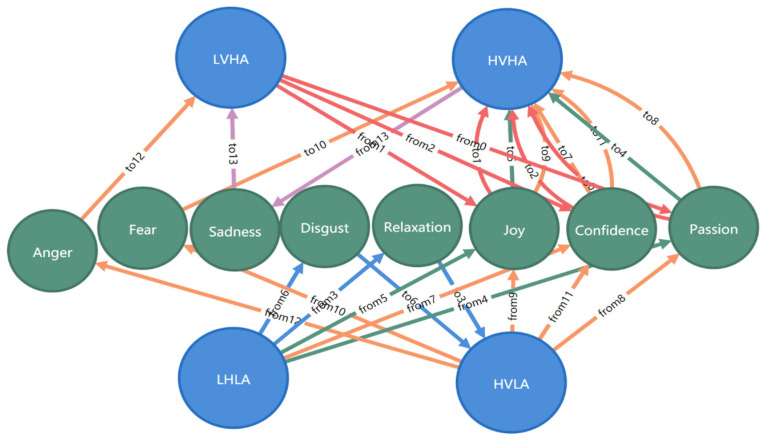
A graph for the relationship between music video types and emotion change in the valence arousal model.

It was relatively difficult to analyze single emotions, so we analyzed the relationship between music video types and emotion change in the valence arousal model, as is shown in Table 8. In addition, in order to facilitate observation, the relationship graph was drawn, as is shown in Figure 11. The music videos of passion, joy, and confidence could improve the emotion of high arousal from a low valence state to a high valence state, with the highest promotion rate being 92%. The music videos of disgust and relaxation could improve the emotion of low arousal from a low valence state to a high valence state, and the improvement rate reached 50%. The music videos of joy and passion could change the emotion from low to high arousal by an improvement rate of 68%. The music videos of confidence, joy, passion, fear, and anger could improve the emotion from low arousal to high arousal, with the highest promotion rate being 81%. The music videos of anger and sadness could reduce the emotion of a high valence state to a low valence state, and the probability reached 48%.

In addition, we designed an experiment to verify music therapy. We mainly used Mindlink EEG equipment to verify the relationship between music influences on emotion and mood changes using music therapy. We used MindAsset’s MindLink EEG device to collect EEG signals and carried out a music therapy experiment under the condition of ensuring high concentration among 13 participants. The bandwidth of EEG acquisition was 3–100 Hz, the sampling rate was 512 Hz, and the channel of EEG was FP1. The experimental process was consistent with the DEAP dataset, and emotional feedback was obtained by listening to music. The types of music emotions used in the experiment were consistent with those in the previous study. We used the self-rating method to record emotional labels, and also recorded music labels used in the experiment. Finally, the experimental results were analyzed. It was found that anger and fear music led to the occurrence of the fear emotion, and the probability wa 80%. Joy and confidence music could improve most of the emotions to joy and passion emotions, and the probability was more than 67%. Sadness type music reduced emotions to relaxation and sadness emotions, and the probability was more than 67%.

## 4. Discussion

In our test, the emotion “intimacy” recognition accuracy was very low for EEG signals. This was due to the fact that “intimacy” was more easily mistaken as the emotion “surprise,” which was due to their very similar semantic distances. By observing the position of these two emotions in the two-dimensional emotional model, we found that they were very close, so the two emotions were mistaken for each other. Moreover, the emotions “sadness” and “relaxation” seemed to involve the same situation in the music emotion classification processing. The audio characteristics of these two kinds of music revealed that each audio feature was very similar, so the two kinds of music were more prone to recognition errors. In the analysis of the relationship between music types and mood changes, positive music usually improved emotions, while negative music made emotions worse. However, we found that disgust music could also improve the mood from low valence to high valence, which was an interesting discovery. In fact, in music therapy, users can play positive music to achieve a healing effect, but the relationship between music and emotional changes needs to be further studied.

In the music classification, we combined emotional elements with music classification to build an emotional music dataset, so as to realize the emotional classification of the music. After obtaining the music type emotional labels, we tracked the people’s emotion changes by music influence to further discuss the potential association between them. In the future, we will expand the types of music emotions to enrich the music database for further experiments.

In this study, we used an EEG to identify 20 emotion types, and calculated the minimum number of channel selections under the requirement of a guaranteed accuracy rate. We hope this research can be widely used in portable applications, and not only for the medical field. It will more accessible to users. So, in our testing, in addition to using the well-known open datasets to discuss the recognition accuracy for emotions, we also used Muse 2, Emotiv insight, and Mindlink to gather EEG signals and test the emotional recognition accuracy. In our previous study, for different types of channel selections, the average recognition rate could reach 93.5% [31]. At the core of improving the accuracy of multiple types of emotion calculations is semantic analysis for classification. So, in this paper, we focused on discussing the music therapy effectiveness for the further analysis of the emotional music response, which helped users to relieve bad moods. In the future, we will consider combining with some other kinds of portable collection devices to test a wide applicability and to further improve the accuracy.

## 5. Conclusions

In this paper, we performed deep analysis of music therapy for emotion changes by calculation of the EEG and audio signals effects. The experimental results of the EEG emotion semantic recognition showed that 20 kinds of emotions were successfully identified using 32 channels, and good results were achieved in each frequency band. Therefore, the EER model has good performance for EEG emotional semantic analysis. The average accuracy for each of the four frequency bands was above 94%, which showed that the four bands could effectively identify emotions. Moreover, in order to optimize the EEG channels for mobile application, five channels (P7, FC1, PZ, T7, and CP2) were used for emotion recognition, and had an average accuracy of more than 90%.

The experimental results of emotion classification based on the audio signal showed that eight kinds of music were successfully classified by using nine kinds of audio features, and the average accuracy was 94%. Therefore, the MEC model has good performance for audio signal processing, and it analyzed the classification of each of the audio features, where those using just MFCC features could achieve 90% accuracy.

In the final study, we also discussed the correlations between music types and mood changes. It was found that joy and confidence music could improve most emotions. Joy, passion, confidence, and relaxation music could cause positive emotions, such as surprise and intimacy. Fear, anger, disgust, and sadness music could cause mania, anxiety, disgust, tiredness, and other negative emotions. The EER model provided an objective means for emotion recognition in music therapy, and the MEC model provided a music emotion classification method, which could better improve the effect of music therapy in accordance with to the relationship between music category and emotional change.

## Figures and Tables

**Table 1:** EEG signals decomposition.

Frequency Band	Frequency Range (Hz)	Decomposition Level
*δ*	1–4	A4
*θ*	4–8	D4
*α*	8–16	D3
*β*	16–32	D2
*γ*	32–64	D1

**Table 2:** Notations for EER algorithm.

Parameters	Definition
ES	Raw EEG signal
*TPTR*	Denoising threshold method
*WNAME*	Wavelet function used
WDS	Wavelet denoised signal
*WDL*	Wavelet decomposition level
*FBS*	Frequency bands signal
*AP*	Average PSD
−*t*	The used kernel was RBF
cmd	Training model parameter list
Acc	Classification accuracy of SVM

**Table 3:** Statistics of emotional music clip.

Emotion Types	Number of Songs	Number of Clips
Anger	40	823
Confidence	15	198
Disgust	27	460
Fear	46	802
Joy	69	931
Passion	45	716
Relaxation	60	1079
Sadness	54	923

**Table 4:** Extracted audio feature list.

Types	Features	Dimension
Timbre	MFCC Spectral Centroid Spectral Flux	13
Temporal	Zero Crossings	1
Intensity	Root Mean Square	1
Rhythm	Beat Per Minute (BPM) BPM Intensity	1
Harmony	Major and Minor mode	1
Other	Roughness	1

**Table 5:** Parameters in Algorithm 2.

Parameters	Definition
*MS*	Music signal
*SN*	Number of signal segments
fix	A truncation function
SST	Segments start point
SEN	Segments end point
*SS*	Signal after segmentation
MFV	Music feature vectors
*−t*	The used Kernel was RBF
cmd	Training model parameter list
Acc	Classification accuracy of SVM

**Table 6:** Extraction audio features statistics.

Music Types	Spectral Centroid	Spectral Flux	Zero Crossings	RMS	BPM	BPM Intensity	Major Minor	Roughness
Anger	1,585,362	102,555	48,650	0.225	126	0.308	−1.352	1,501,663
Confidence	1,791,002	109,347	43,150	0.259	123	0.42	−0.852	1,119,187
Disgust	1,727,352	113,576	54,299	0.234	132	0.321	−0.666	2,010,873
Fear	1,523,582	101,266	42,290	0.242	131	0.326	−1.311	1,494,369
Joy	1,586,149	102,634	42,128	0.238	126	0.411	−0.874	1,278,455
Passion	1,599,357	99,364	41,068	0.234	124	0.458	−1.114	983,718
Relaxation	810,791	51,005	19,871	0.188	122	0.205	−1.25	545,603
Sadness	1,142,621	63,860	27,318	0.2	119	0.193	−1.367	731,995

**Table 7:** Music video types and emotion relationships.

Original Emotion Types	Music Video Types	After Music Therapy	Probability (%)
Concentration	Confidence	Surprise	50
Disgust	Confidence	Surprise	50
Fear	Confidence	Surprise	41.67
Fear	Joy	Surprise	47.06
Disgust	Joy	Intimacy	45.45
Manic	Joy	Surprise	44.44
Trust	Joy	Surprise	44.44
Intimacy	Sadness	Fear	46.15

**Table 8:** Video types and emotions relationship in valence-arousal model.

Original Emotion Types	Music Video Types	After Music Therapy	Probability (%)
LVHA	Passion	HVHA	80
LVHA	Joy	HVHA	92.68
LVHA	Confidence	HVHA	78.95
LVLA	Confidence	HVHA	66.67
HVLA	Passion	HVHA	70.59
HVLA	Joy	HVHA	81.40
HVLA	Fear	HVHA	54.55
HVLA	Confidence	HVHA	60
HVLA	Anger	LVHA	48.15
LVLA	Joy	HVHA	68.18
LVLA	Passion	HVHA	50
LVLA	Relaxation	HVLA	50
LVLA	Disgust	HVLA	47.37
HVHA	Sadness	LVHA	41.18

## Data Availability

Publicly available dataset was analyzed in this study. This data can be found here: (http://www.eecs.qmul.ac.uk/mmv/datasets/deap/).
